# P02.114. Improvement in postural response as a possible mechanism for decrease in falls with vitamin D

**DOI:** 10.1186/1472-6882-12-S1-P170

**Published:** 2012-06-12

**Authors:** A Peterson, F Horak, M Martina

**Affiliations:** 1 Oregon Health & Science University/Portland VA, Portland, USA

## Purpose

To evaluate the relationship between balance performance and vitamin D in persons with Parkinson’s disease (PD). Postural instability is one of the four cardinal features of PD. Balance problems and falls are a major source of morbidity and mortality late in the disease largely because there are no effective treatments. Vitamin D supplementation reduces falls and reduces sway in quiet stance in elderly fallers.

## Methods

Subjects underwent a battery of 5 balance tests on the Neurocom posture platform, were tested for a number of possible confounders: Parkinson’s severity (motor UPDRS), cognitive function (trails A & B, MMSE, verbal fluency, and clock draw), dyskinesias (mAIMS), and had serum drawn for vitamin D levels. A Pearson’s product-moment correlation was performed to investigate the association between balance measures and vitamin D levels (correcting for UPDRS score).

## Results

The most striking correlation was found between the vitamin D levels and the automatic posture response to backwards translations. The strongest correlations with vitamin D levels were between postural response strength asymmetry and stance weight asymmetry. Correcting for the UPDRS, the correlation coefficients for vitamin D and strength symmetry were 0.41 (p=0.01), 0.34 (p=0.03), and 0.32 (p=0.05) for small, medium, and large perturbation, respectively. The correlation coefficients for vitamin D and weight symmetry were similar at 0.32 (p=0.05), 0.42 (p=0.01), 0.30 (p=0.07).

## Conclusion

Automatic postural responses are crucial for avoiding falls. Asymmetry of automatic postural responses puts subjects at increased risk for falls especially when weight shifts are necessary, such as when walking or reaching for an object. It is possible that vitamin D may have a specific effect on neural control of postural responses and this supports further intervention studies to see if vitamin D supplementation decreases fall risk in patients with PD.

